# Virtual reality and sports performance: a systematic review of randomized controlled trials exploring balance

**DOI:** 10.3389/fspor.2025.1497161

**Published:** 2025-04-29

**Authors:** Ida Cariati, Roberto Bonanni, Pierangelo Cifelli, Giovanna D'Arcangelo, Elvira Padua, Giuseppe Annino, Virginia Tancredi

**Affiliations:** ^1^ Department of Systems Medicine, “Tor Vergata” University of Rome, Rome, Italy; ^2^ Department of Biotechnological and Applied Clinical Sciences, University of L’Aquila, L’Aquila, Italy; ^3^ Centre of Space Bio-Medicine, “Tor Vergata” University of Rome, Rome, Italy; ^4^ Department of Human Sciences and Promotion of Quality of Life, San Raffaele Telematic University of Rome, Rome, Italy

**Keywords:** virtual reality, sport performance, balance, athletes, players, training, sports, RCTs

## Abstract

**Introduction:**

Our systematic literature review aimed to select randomized controlled trials (RCTs) in which virtual reality (VR) has been used in athletes or players to evaluate the effectiveness of this technology in gaining performance.

**Methods:**

In accordance with PRISMA guidelines, a systematic literature search was conducted in the MEDLINE, Scopus and Web of Science databases using the keyword set [(Virtual reality) OR (VR)] AND [(Athletes) OR (Players)] AND [(Performance) OR (Balance)]. Peer-reviewed articles published within the last ten years in English and open access were included. The methodological quality of the articles was assessed using the Jadad scale, while the eligibility criteria were evaluated using the PICOS approach.

**Results:**

Specifically, six RCTs were selected, one of which scored 5/5 on the Jadad scale, four scored 3/5 and one scored 2/5. Importantly, five RCTs found a positive influence of VR on performance in terms of balance, stability, sprinting, jumping, neurocognitive function, reaction time and technical skills, while only one RCT found no difference in these parameters.

**Discussion:**

In conclusion, the results included in our systematic review showed that VR seems to have a positive effect in improving sports performance. However, the heterogeneity of the studies did not allow for a comparison of the data to clarify the relevance of VR technology in performance, suggesting the need for in-depth investigations to confirm its efficacy in sports.

## 1 Introduction

Virtual reality (VR) has taken on a crucial role in everyday life, becoming commonly used in many educational and recreational settings, as well as in surgery and rehabilitation (1–3). Indeed, VR technology is known to improve balance, gait, posture and reduce falls in the elderly and in patients with Parkinson's or stroke, suggesting an important role in improving physical performance (4–9).

Noteworthy, in recent years VR has also been used in sports in association with traditional training, to improve performance (10). In this context, Harrison et al. demonstrated that VR interventions using head-mounted displays are useful in significantly reducing anxiety levels and increasing self-esteem in female football players, although performance was not directly affected by VR (11). On the other hand, Novak and colleagues analyzed the effects of a 5-min VR training programme on dynamic balance in tennis players, concluding that such technology has the potential to influence physical performance (12). Interestingly, Bedir and Erhan conducted a semi-experimental study analyzing the effect of VR-based training programmes on the shooting performance and imaginative abilities of athletes (13). The authors divided 14 curling, 13 bowling and 7 archery athletes into a control group, a group subjected to 2D video-guided progressive muscle relaxation exercises, and a group subjected to 3D video-guided progressive muscle relaxation exercises applied via VR glasses. After a period of 4 weeks, athletes trained with VR technology showed significant improvements in shooting performance, suggesting an important role of VR in neurocognitive function (13). In addition, Reneker et al. conducted a semi-experimental study to evaluate the effects of virtual immersive sensorimotor training on injury incidence rates and on-field performance in football athletes from two different universities (14). Specifically, 78 participants recruited at one university received virtual immersive sensorimotor training consisting of nine exercises involving vestibular, visual, oculomotor, cervical neuromotor control, movement co-ordination, postural and balance exercises, twice a week for 6 weeks; while the control group consisted of 52 participants recruited at the second university who did not undergo virtual immersive sensorimotor training. Importantly, an improvement in tests of cervical neuromotor control and balance in the intervention group was observed, as well as a positive association between one of the virtual trainings and performance on the pitch, suggesting the potential of immersive VR training in improving football performance (14).

Overall, some evidence proposes VR technology as a useful contribution to sports training due to the possibility of transferring virtually learned experiences to the real world. Harris et al. point out that the success of VR simulations depends on their fidelity in reproducing real-world conditions, not only visually, but also psychologically, affectively and ergonomically (15). Adequate simulation authenticity is crucial for generating realistic behaviors that can significantly improve athletes' performance, including balance, reaction time, motor skills, and sport-specific technical skills (16). However, the effectiveness of VR in improving physical performance in sport needs substantial further investigation as limited evidence of its effectiveness is available. Indeed, systematic reviews published to date have often included studies with heterogeneous experimental designs, both observational and interventional, making it difficult to draw firm conclusions. Based on these premises, our systematic review aims to (i) identify randomized controlled trials (RCTs) related to the use of VR in improving sports performance and (ii) evaluate the effectiveness of this technology in gaining performance in athletes and/or players, with a focus on balance, a crucial parameter for many sports disciplines. In fact, improving balance not only directly affects motor skills but also plays a crucial role in injury prevention (17), making it a strategic goal in many sports training routines. Therefore, our review is distinguished by the exclusive inclusion of RCTs, ensuring a higher level of evidence and a more rigorous evaluation of the effectiveness of VR. This approach could provide a clearer picture on the applicability of VR as an additional tool to traditional training, determining whether it can be a valuable support for improving athletic performance.

## 2 Materials and methods

This systematic review investigating the influence of VR in improving sports performance in athletes and/or players complies with preferred recording items for systematic review and meta-analysis (PRISMA) guidelines to promote transparency and reproducibility (18). The study protocol was established prior to conducting the systematic literature search and was registered in the open science framework (OSF) repository with the doi: 10.17605/OSF.IO/QXD5G to ensure accessibility of the methodology and improve the integrity of the review process.

### 2.1 Eligibility criteria, source information and search strategy

RCTs on the use of VR in sports training to improve performance, especially balance, were searched. A systematic literature search was conducted on 26 July 2024 using the full set of keywords [(Virtual reality) OR (VR)] AND [(Athletes) OR (Players)] AND [(Performance) OR (Balance)]. Three electronic databases, MEDLINE, Scopus and Web of Sciences, were consulted, applying filters to narrow the results and selecting peer-reviewed open access articles published in the period 2014–2024 in English. Only RCTs were included in the final selection, while systematic and non-systematic reviews, meta-analyses, non-randomized experimental studies and non-experimental studies were excluded.

Study eligibility was assessed using the PICOS approach (population, intervention, comparison, outcome and study design) (19), with inclusion and exclusion criteria shown in Table 1.

**Table 1:** Selection criteria by PICOS approach.

Category	Inclusion criteria	Exclusion criteria
Population	Athletes, players	Non-Athletes, non-players
Intervention	Use of VR technology associated with training	Absence of the use of VR technology associated with training
Comparison	Active or non-active control group, traditional training group without VR technology	Absence of an active or non-active control group or a traditional training group without VR technology
Outcome	Measurement of sports performance, especially balance	Lack of baseline and/or follow-up data
Study design	RCT	All other study designs

VR, virtual reality; RCT, randomized control trial.

### 2.2 Selection process, data collection and quality control

The initial keyword search and methodological quality assessment were performed by two experienced researchers. All articles retrieved from the selected databases were loaded into Rayyan's reference management software and were independently screened for duplicates. Subsequently, a screening for titles and abstracts was conducted to exclude articles unrelated to the topic. A third researcher clarified any doubts about article selection. All RCTs whose methodological quality was examined using the Jadad scale were selected.

## 3 Results

**Figure 1:**
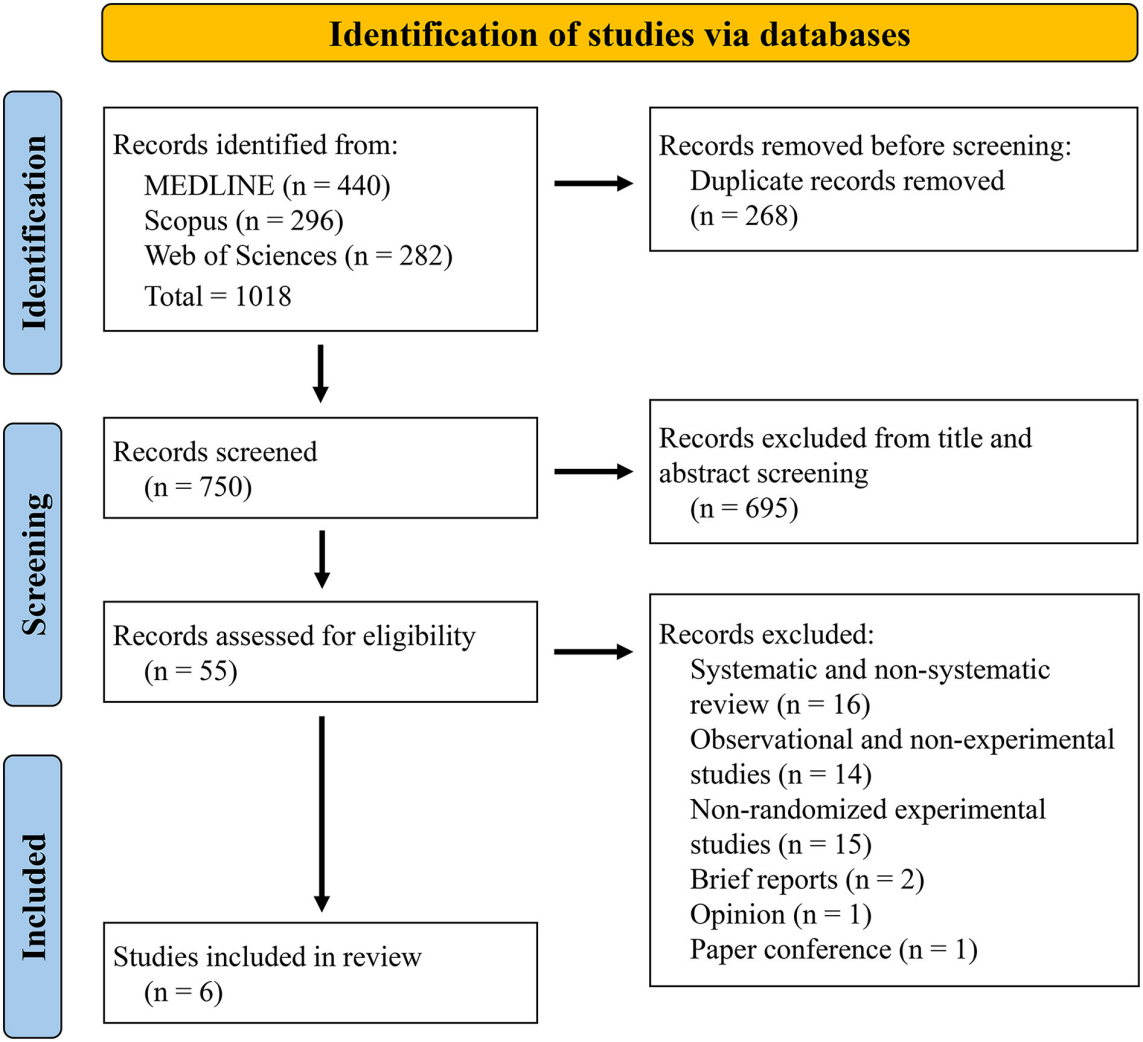
PRISMA flowchart of the study.

The initial search yielded a total of 1,018 articles, of which 440 were from MEDLINE, 296 from Scopus and 282 from Web of Sciences. After importing all articles into the Rayyan software, 268 records were excluded as duplicates. Subsequent screening by title and abstract on 750 remaining records excluded 695 articles as inconsistent with the topic of our systematic review. Notably, many of these studies did not investigate the effects of VR on sports performance, especially on balance, which is the focus of our systematic research. Some articles treated VR in different contexts, such as cognitive learning or rehabilitation, without exploring the direct impact on motor performance and balance improvement. Other studies were excluded because the study population did not include athletes and/or players, who are the target of our review. In addition, some articles did not provide sufficient details about the VR technology used, making it difficult to assess its consistency with the objectives of our research. Of the 55 articles selected for eligibility, 16 were eliminated as systematic or non-systematic reviews, 14 as non-experimental studies, 15 as non-randomized experimental studies, 2 as short reports, 1 as an opinion and 1 as a paper conference. Therefore, a total of 6 RCTs were included in the systematic review, as shown by the study flow chart in Figure 1.

### 3.1 Characteristics of included studies and quality assessment by Jadad scale

The quality of the 6 RCTs included in our systematic review was analyzed using the Jadad scale by assigning a score from 0 to 5 to each individual study to assess the randomization methods used, the level of blindness and the appropriate description of participants who did not reach follow-up. Overall, one study scored 2, four studies scored 3 and one study scored 5, as shown in Table 2.

**Table 2:** Methodological quality assessment by Jadad scale.

Reference	Is the study described as randomized?	Is the study described as double-blind?	Is there a description of the lost at follow-up?	Is the randomization appropriate?	Is blindness appropriate?	Total score
(20)	1	1	1	1	1	5
(21)	1	0	1	1	0	3
(22)	1	0	1	1	0	3
(23)	1	0	1	1	0	3
(24)	1	0	1	1	0	3
(25)	1	0	1	0	0	2

The study by Nambi et al. was a double-blind RCT to identify and compare the effects of isokinetic training and VR training on the sports performance of 45 university football players with chronic low back pain. The randomization of the participants into 3 groups was carried out by a person not involved in the data collection, and the simple random table method was adopted maintaining a 1:1:1 ratio. Blinding of the study was appropriate because the subject and therapist who assessed the outcomes at follow-up were blinded, although it was not possible to blind the treating therapist (20).

In 2021, Shousha and colleagues published the results of an RCT to compare the effect of VR training using Wii Fit Plus games with Biodex balance training in 90 male football players with ankle instability caused by grade II lateral sprain randomized into 3 groups. The randomization is appropriately described and there is a description of the subjects lost to follow-up. However, the study is not described as double-blind (21).

In 2023, Mohammadi et al. published the results of a single-blind randomized paired study to compare the effects of traditional and VR training on the subjective sensation of instability and balance in basketball players suffering from functional ankle instability. A block randomization was performed using random allocation software that divided the 54 players into two groups (22). In the same year, the same authors published the results of a single-blind paired study to evaluate the effect of VR training with Wii Fit Plus on the neurocognitive function of basketball athletes with functional ankle instability. Randomization was performed using random allocation software that divided 50 athletes into two groups (23). Both studies reported on the number of individuals at follow-up, but neither was conducted in a double-blind manner.

In 2023, Wang and colleagues published the results of a randomized controlled crossover study designed to examine the influence of boxing in VR on cardiopulmonary parameters and lactic acid levels in 50 healthy male participants trained regularly randomized into two groups. Randomization for group assignment was performed using special software and information on individuals present at follow-up was reported, although the study was not double-blinded (24).

Finally, the RCT by Rusmanto et al. evaluated the effectiveness of VR in increasing the level of sports engagement and improving technical skills in 40 junior football athletes. Although the authors found no drop-outs throughout the duration of the RCT, they did not provide additional details on the randomization of the young footballers into the two experimental groups (25).

### 3.2 Summary of results

The double-blind RCT by Nambi et al. (20) included 45 male University football players aged 18–25 years with chronic low back pain and pain intensity measured by visual analogue scale (VAS) between 4 and 8, randomized into 3 groups. Specifically, 15 participants performed isokinetic training (IKT) 5 days/week for 4 weeks while standing upright, with knees flexed 15° and a trunk range of motion of 10° in extension and 80° in flexion, using an isokinetic dynamometer. The exercises were performed at an angular speed of 60 degrees/second, 90 degrees/second and 120 degrees/second with 15 repetitions of 3 sets and a 30-s rest between each 60-s rhythm. The virtual reality training (VRT) group received 30-min balance training, 5 days/week for 4 weeks with the ProKin system PK 252 N Technobody focusing on balancing the core stability muscles. The training was provided in a seated position and consisted of a shooting game in which the subject could move the trunk back and forth and left and right, performing all six spinal movements within the limits of pain. The difficulty of the tasks gradually increased as the subject performed more muscle movement. Finally, the control group was prescribed conventional balance training for the core muscles for 10–15 repetitions per day, 5 days/week for 4 weeks, characterized by isotonic and isometric exercises for the abdominal muscles, deep abdominals and back muscles. VAS scores at baseline were not significantly different between the groups, while a significant reduction in pain was found in the IKT and VRT groups at 4 weeks, 8 weeks and 6 months follow-up. Interestingly, the VRT group showed a greater reduction in pain and better well-being than the other participants. Greater benefits were also found in the VRT group in improved sports performance. Particularly, the performance of 40 m sprint, 4 × 5 m sprint and submaximal shuttle running was evaluated, finding significant differences between the groups after 4 weeks, 8 weeks and 6 months of follow-up, with a greater effect for the VRT group. Finally, the performance of countermovement jump (CJ) and squat jump (SJ), performed 4 times with 30 s of rest, were measured at baseline and at follow-up times, observing significant differences between groups from week 4 onwards. Again, better results were found at 6 months in the VRT group than in the IKT and control group.

In the RCT by Shousha et al. (21), 90 male football players aged 12–16 years with ankle instability caused by grade II lateral ankle sprain were randomized into three groups. The control group (*n* = 30) underwent a training programme according to the guidelines for proprioceptive and balance rehabilitation based on performing progressive positional activities on one limb with eyes open or closed for 60 min/day, 3 times/week for 3 months. Progression from one level to the next involved completing the previous exercise without errors, which included stabilizing with the contralateral limb, bending the trunk more than 30°, removing the hands from the hips, and resting the non-weight-bearing extremity on the weight-bearing extremity. In addition, balance exercises were performed using a sliding board, trampoline and donut hall. The VR group (*n* = 30) underwent the same training programme according to the guidelines for proprioceptive and balance rehabilitation as the control group for 30 min/day, 3 times/week for 3 months, in addition to 30 min of VR, administered via the Nintendo Wii Fit Plus device. This included a programme of strength exercises, such as lunges, one-leg twisting, rowing squats, one-leg extension and lateral leg lifts, as well as balance exercises, such as tight rope walk, ski slalom, snowboard slalom, soccer heading and table tilt. Finally, the biodex balance training (BBT) group (*n* = 30) performed the proprioceptive rehabilitation and balance training programme for 30 min/day, 3 times/week for 3 months, in addition to 30 min of biodex balance system (BBS) training for balance and postural stability assessment. At the end of the training period, all participants underwent assessment of overall stability index (OASI), antero-posterior stability index (APSI), medio-posterior stability index (MLSI), and cumberland ankle instability tool (CAIT), the values of which were compared with measurements taken at baseline. Interestingly, a significant reduction in OASI, APSI and MLSI was observed in all groups after 3 months of treatment, with lower values in the VR and BBT groups. In agreement, a significant increase in CAIT was observed at post-treatment, with a greater improvement in the VR and BBT groups than in the control group. Noteworthy, no significant differences were found between the VR and BBT groups for the measured variables.

The paired RCT by Mohammadi et al. (22) involved 54 basketball athletes aged 19–25 years with functional ankle instability (FAI) randomized into two groups, a control group (*n* = 27) and a VR group (*n* = 27). All participants performed 4-week, 3-day/week training for 12 sessions. Specifically, the athletes in the VR group used the Nintendo Wii Fit Plus device to perform strengthening exercises, including single leg extension, sideways leg lift, single leg twist and rowing squat, and balance training games, such as soccer heading, ski slalom, tight rope walk and table tilt. Athletes in the control group performed traditional exercises such as plantar flexion and dorsiflexion. In addition, they performed training consisting of three sets with 10 repetitions for each movement with the Thera-Band, as well as balance board exercises in which, during the first week, the board was moved backwards; in the second week, the left and right edges were mobilized against the floor, and in the last two weeks, circular movements were performed. These exercises, during which the board did not touch the floor, lasted 15 s, with 10 s of rest, and were repeated 10 times. The subjective sense of instability and balance was measured with the CAIT and the star excursion balance test (SEBT) respectively and assessed before and after the test and at one month follow-up. At baseline, no significant differences were found in the CAIT score between the two groups. In contrast, significant differences in the posterior and postero-medial directions of the SEBT and CAIT score were found for the involved limb at post-test, as well as in the posterior direction of the SEBT and CAIT score at one month follow-up, with slightly better performance in the VR group.

In the same year, Mohammadi et al. (23) published another single-blind matched RCT on 50 basketball athletes aged between 20 and 30 years randomized into two groups: a VR group consisting of 25 athletes with unilateral FAI, and a control group that included 25 basketball athletes without FAI and no intervention. Specifically, the athletes in the VR group performed a 5-min warm-up on an exercise bike and training with the Nintendo Wii Fit Plus device, 3 times/week for 12 sessions, performing balance exercises, such as ski slalom, soccer heading and tight rope walk, as well as strengthening games with coordinated upper and lower limb movements, including sideways leg lift, single leg extension, single leg twist and rowing squat. At the beginning and the end of the training period, the Deary-Liewald reaction time task (DLRT) was used to assess each athlete's neurocognitive function and information processing capacity by measuring through the presentation of the “X” sign on a computer monitor both detection, in terms of simple reaction time (SRT), and identification, in terms of choice reaction time (CRT). Specifically, the tests were performed randomly at 1-min intervals and each subject performed each test three times, recording reaction times and errors. Interestingly, the impairment of neurocognitive function observed in the VR group at baseline was neutralized by the 12 training sessions, with a significant improvement in SRT and CRT compared to the control group. In contrast, the measures taken at baseline and at the end of the study on the control group remained unchanged.

In 2023, Wang et al. (24) conducted a balanced randomized controlled crossover study in which 50 physically active healthy adults aged 40–50 years with at least 3 years of exercise experience were randomly assigned to VR (*n* = 25) and non-VR (*n* = 25) groups. All participants performed, on separate days with approximately 48 h apart, six supine boxing exercise protocols supported by a hanging frame involving a combination of two set-intervals of 0–40 s and three repetitions-intervals of rest time set at 0 s, 1/3 s or 2/3 s, for a total of 720 punches. In addition, all participants were equipped with a VR visor and controller (PICO Neo 3 Pro) that projected a game scene consisting of self-selected combat environments and opponent avatars for the VR group only. Heart rate, oxygen uptake and ventilation volume were monitored continuously during the training, in addition to lactic acid levels measured immediately after the last repetition, as well as 3 min, 5 min, 10 min, 20 min and 30 min after the last repetition. Although significant differences were found between the exercise protocols within each group, no differences associated with the VR intervention were observed in cardiopulmonary measures, in terms of which mean heart rate, oxygen consumption, respiratory quotient, maximal ventilation volume, oxygen pulse, excess post-exercise oxygen consumption (EPOC) and lactic acid.

Finally, in the RCT by Rusmanto et al. (25), 40 junior football athletes aged 16–20 years were randomized into an experimental group (*n* = 20) and a control group (*n* = 20). All athletes followed traditional football training of 60 min/day, 3 times/week for 12 weeks. In addition, participants in the experimental group performed a virtual football training programme with shooting and passing actions, 3 sets of 15 min/day with 5 min rest between two consecutive sessions. Technical football skills were measured by means of the shooting test to assess the athlete's shooting performance, in which the participants performed 14 shots on target over two series, and the pass test to determine the athlete's passing ability by executing passes over 10 m and a width of one metre. Important, a significant increase in the technical skills measured after 12 weeks of training was observed in the experimental group, while no improvement was noted for the control group.

Table 3 summarizes the 6 RCTs included in our systematic review, with detailed information on the study population, the type of intervention, the VR instrumentation used and the main study results.

**Table 3:** A schematic representation of the results of the RCTs included in the study.

Reference	Study population	Intervention	VR instrumentation	Results
(20)	*n* = 45 University football players with chronic low back pain - *n* = 15 control group; age (years): 20.78 ± 1.6; males - *n* = 15 IKT group; age (years): 20.23 ± 1.6; males - *n* = 15 VRT group; age (years): 21.25 ± 1.2; males	- Control group: conventional balance training, 10–15 repetitions per day, 5 days/week for 4 weeks - IKT group: 3 sets of 15 repetitions of isokinetic training 5 days/week for 4 weeks, with a 30-second rest between each 60-second rhythm - VRT group: balance training for 30 min, 5 days/week for 4 weeks	ProKin system PK 252 N Technobody, pelvic module balance trunk MF, Italy	- Reduction in VAS scores in all groups at 4 weeks, 8 weeks and 6 months, with most significant results for the VRT group - Improvement in 40 m sprint, 4 × 5 m sprint and submaximal shuttle running performance in all groups at 4 weeks, 8 weeks and 6 months, with more significant results for the intervention groups and especially for the VRT group - Improved CJ and SJ performance in all groups at 4 weeks, 8 weeks and 6 months, with most significant results for the VRT group at 6 months
(21)	*n* = 90 adolescent football athletes with chronic ankle instability - *n* = 30 control group; age (years): 15.23 ± 0.84; males - *n* = 30 VR group; age (years): 15.74 ± 0.95; males - *n* = 30 BBT group; age (years): 15.32 ± 0.92; males	- Control group: proprioceptive and balance rehabilitation training (progressive positional activities on one limb with eyes open or closed, stabilization with the contralateral limb, bending of the trunk by more than 30°, removal of hands from hips, resting of the non-weight-bearing extremity to the weight-bearing extremity) for 60 min, 3 times/week for 3 months - VR group: proprioceptive and balance rehabilitation training for 30 min, 3 times/week for 3 months, in addition to 30 min of VR with a strength exercise programme (lunges, one-leg twist, rowing squat, one-leg extension, lateral leg lift) and balance exercises (tight rope walk, ski slalom, snowboard slalom, soccer heading, table tilt) - BBT group: proprioceptive and balance rehabilitation training for 30 min, 3 times/week for 3 months, in addition to 30 min of BBS training	Nintendo Wii Fit Plus	- Significant reduction of OASI, APSI and MLSI in all groups after 3 months of treatment, with better results for the VR and BBT groups - Significant increase in CAIT in all groups after 3 months of treatment, with better results for the VR and BBT groups - Statistical analysis revealed significant differences between the control group and the intervention groups, but not between VR and BBT groups
(22)	*n* = 54 basketball athletes with FAI - *n* = 27 control group; age (years): 21.78 ± 2.29; males - *n* = 27 VR group; age (years): 22.04 ± 2.10; males	- Control group: 12 traditional training sessions (plantar flexion, dorsiflexion, inversion and eversion with Thera-Band and balance board exercises), 3 days/week for 4 weeks - VR group: 12 VR sessions, 3 days/week for 4 weeks, with strengthening exercises (single leg extension, sideways leg lift, single leg twist, rowing squat) and balance training games (soccer heading, ski slalom, tight rope walk and table tilt)	Nintendo Wii Fit Plus	- Significant increase in posteromedial and posterior SEBT score and CAIT score in the post-test for the involved limb - Significant increase in posterior SEBT score and CAIT score at one month follow-up for the involved limb - Statistical analysis found better performance for the VR group than for the control group, albeit with a small effect size
(23)	*n* = 50 basketball athletes - *n* = 25 control group without FAI; age (years): 22.16 ± 1.95; males - *n* = 25 VR group with unilateral FAI; age (years): 21.56 ± 2.31; males	- Control group: no intervention - VR group: 12 VR sessions, 3 days/week for 4 weeks, with balance exercises (ski slalom, soccer heading and tight rope walk) and strengthening games with coordinated movements of the upper and lower limbs (sideways leg lift, single leg extension, single leg twist and rowing squat)	Nintendo Wii Fit Plus	- Significant improvement in neurocognitive function in terms of SRT and CRT in the VR group after 12 training sessions - No significant change in neurocognitive function in the control group between baseline and the end of the study
(24)	*n* = 50 physically active adults - *n* = 25 VR group: age (years): 45.28 ± 1.31; males - *n* = 25 non-VR group: age (years): 44.92 ± 1.50; males	Six sets of 120 boxing exercises with two rest periods at series intervals (0–40 s) and three at repetition intervals (0 s, 1/3 s or 2/3 s), in supine position	- VR visor and controller (PICO Neo 3 Pro, PICO Immersive Pte, USA) worn by both groups - Game scene presented only in the VR group, with self-selected combat environments and opponent avatars	No significant differences in cardiopulmonary measures, including mean heart rate, oxygen consumption, respiratory quotient, maximum ventilation volume, oxygen pulse, EPOC and lactic acid
(25)	*n* = 40 junior male football athletes aged 16–20 years - *n* = 20 control group - *n* = 20 experimental group	- Control group: traditional football training, 60 min/day, 3 times/week for 12 weeks - Experimental group: traditional football training, 60 min/day, 3 times/week for 12 weeks, in addition to a virtual football VR programme with shooting and passing actions for 15 min/day, 3 times/day with 5 min rest	No information on the type of VR instrument used	- Significant increase in technical skills, measured by shooting test and pass test, in the experimental group after 12 weeks of training - No significant difference in technical ability was found in the control group between the beginning and end of training

RCTs, randomized controlled trials; IKT, isokinetic training; VRT, virtual reality training; VAS, visual analogue scale; CJ, countermovement jump; SJ, squat jump; VR, virtual reality; BBT, biodex balance training; BBS, biodex balance system; OASI, overall stability index; APSI, antero-posterior stability index; MLSI, medio-posterior stability index; CAIT, cumberland ankle instability tool; FAI, functional ankle instability; SEBT, star excursion balance test; SRT, simple reaction time; CRT, choice reaction time; EPOC, excess post-exercise oxygen consumption.

## 4 Discussion

Our systematic literature review selected 6 RCTs in which the study population followed a traditional training programme implemented with VR technology to increase sports performance. The synthesis of the results showed that 5 RCTs support the use of VR technology as an effective tool for improving performance and balance (20, 23, 21, 22, 25). Among these, however, the study by Mohammadi et al. found a small effect size, suggesting that the impact of VR in the context of performance might be negligible (22). In contrast, Wang and colleagues found no significant improvements in performance associated with the use of VR, highlighting the presence of contradictory results in the scientific literature and the need for in-depth investigations into the impact of VR in sports performance (24). Nevertheless, the results summarized here overall show a potential effect of VR technology in the performance, particularly in balance, of athletes from different sports disciplines.

The impact of VR technology on balance has been demonstrated in numerous clinical studies conducted on children with cerebral palsy (26), elderly people at risk of falls (27), and stroke or Parkinson's patients (28, 29). In a sports context, VR technology has been widely used to examine the movements and performance of athletes during exercises (16). However, the extraordinary effectiveness of VR in improving balance in the rehabilitation context suggests its potential application also for improving the performance of athletes and players. Indeed, the benefits of VR in supplementing conventional training could be explained through several mechanisms of action. First, VR creates an immersive, multisensory environment that stimulates neuroplasticity (30, 31), potentially promoting faster and more effective motor adaptations. In addition, the ability to receive real-time feedback could allow athletes to refine their motor patterns and immediately correct any errors. An additional benefit is the increased engagement and motivation that VR can induce (32), helping to reduce the perception of effort and improve adherence to the training program. Finally, VR's ability to simulate realistic scenarios and racing situations (33) could offer a unique opportunity to enhance decision-making skills and optimize stress management during competition. In this context, numerous evidences support the effectiveness of VR in improving specific skills, such as decision-making, reaction time reduction and motor control, which could be crucial in a competitive context (34). Therefore, although the use of VR technology for sports performance enhancement represents an emerging study approach, the potential of this innovative technology in the field of sport merits further investigation.

Unfortunately, the evidence available to date supporting the effectiveness of VR in enhancing sports performance, particularly in balance, is still limited and the few studies available do not present a randomized controlled trial design. Conducting high-quality studies with a double-blind randomized experimental design is a necessary step to confirm the effectiveness of a particular intervention. However, the difficulty of conducting a double-blind RCT in the context of using VR technology represents a major limitation to the design and development of high-quality RCTs that should be overcome to ensure adequate reliability and reproducibility of the results obtained. In fact, of the 6 RCTs included in this systematic review, only the study by Nambi et al. (20) achieved the highest score on the Jadad scale; whereas 4 RCTs (21–24) only partially met these criteria with a score of 3 out of 5, and one study (25) only met two items on the Jadad scale with a score of 2 out of 5. Nevertheless, experimental studies with a non-randomized controlled research design have shown promising results on the use of VR in improving sports performance, suggesting its potential application in athletic training (35, 36). Furthermore, interesting results on the use of VR were presented in the context of injury prevention, as well as in rehabilitation, where VR was shown to significantly reduce pain scores in patients with anterior cruciate ligament injury (37, 38). However, it is important to point out that the studies currently available on the role of VR technology in improving sports performance are very diverse, allowing for a qualitative comparison of results but not a quantitative one. Indeed, the diversity in the study population, the sport practised, the intervention administered, and the VR technology used does not allow the development of a meta-analysis aimed at clarifying the relevance of VR in sports performance. Furthermore, the use of VR technology in the sport context seems to be limited only to sub-elite athletes, whereas no evidence of application in elite athletes has been found. This could be due to the ability of current VR technology to induce an improvement in certain skills by conditioning the sub-elite athlete, but without affecting the cardiometabolic parameters that determine sports performance.

To our knowledge, this is the first systematic review of RCTs aimed at investigating the impact of VR technology on sports performance. Although few studies met the inclusion criteria, the comparison of the reported results shows a positive effect of VR on sports performance, especially balance, of healthy athletes, athletes with chronic low back pain or ankle instability. Further high quality studies are needed to investigate the impact of VR in improving performance to promote or discourage the use of this technology in the training of elite athletes.

## 5 Limits of study

Our systematic review has some limitations that affect its generalizability. First, the small number of included studies limits the scope of conclusions. Although the exclusive inclusion of RCTs ensures a high level of methodological quality, the paucity of evidence in this emerging field limits its robustness. However, this gap suggests the need for further randomized trials to strengthen and consolidate the available results. In addition, differences in intervention protocols, VR application modes, and measured parameters complicate direct comparisons between studies. Indeed, variations in the devices used and training modalities can affect the results, as can divergences in performance evaluation criteria and specific measurements, such as balance. Therefore, this methodological variability limits the ability to draw unambiguous and generalizable conclusions and should be considered a preliminary synthesis of the evidence, hoping that new studies will address the gaps identified in this research.

## 6 Conclusions

VR technology has been introduced into every field of work, occupying a prominent role in medicine and education. In the sporting context, VR was initially used to examine movement and sporting performance, but in recent years several evidences have suggested a role for the improvement of performance such as balance, decision-making and response time. However, few papers have evaluated and compared the experimental studies available to date to clarify the impact of VR on sports performance.

Our systematic literature review selected 6 RCTs that investigated the impact of VR technology on athletes’ performance in different sport disciplines, of which only one completely fulfilled the criteria of the Jadad scale. Overall, 5 RCTs showed a positive effect of VR on performance in terms of balance, sprinting, jumping, neurocognitive function, reaction time and technical ability, while one RCT found no influence of VR on performance. The heterogeneity of the included studies, in terms of study population, sport, intervention and technology used did not allow for a comparison of data to determine the relevance of VR technology in improving performance. Therefore, further high quality double-blind RCTs are needed to clarify the impact and utility of VR in sport.

## Data Availability

The datasets presented in this study can be found in online repositories. The names of the repository/repositories and accession number(s) can be found in the article/Supplementary Material.
